# RNA polymerase III-specific general transcription factor IIIC contains a heterodimer resembling TFIIF Rap30/Rap74

**DOI:** 10.1093/nar/gkt664

**Published:** 2013-08-05

**Authors:** Nicholas M. I. Taylor, Florence Baudin, Gudrun von Scheven, Christoph W. Müller

**Affiliations:** ^1^European Molecular Biology Laboratory (EMBL), Structural and Computational Biology Unit, Meyerhofstrasse 1, 69117 Heidelberg, Germany and ^2^UJF-EMBL-CNRS UMI 3265, Unit of Virus Host-Cell Interactions, 38042 Grenoble Cedex 9, France

## Abstract

Transcription of tRNA-encoding genes by RNA polymerase (Pol) III requires the six-subunit general transcription factor IIIC that uses subcomplexes τA and τB to recognize two gene-internal promoter elements named A- and B-box. The *Schizosaccharomyces pombe* τA subcomplex comprises subunits Sfc1, Sfc4 and Sfc7. The crystal structure of the Sfc1/Sfc7 heterodimer reveals similar domains and overall domain architecture to the Pol II-specific general transcription factor TFIIF Rap30/Rap74. The N-terminal Sfc1/Sfc7 dimerization module consists of a triple β-barrel similar to the N-terminal TFIIF Rap30/Rap74 dimerization module, whereas the C-terminal Sfc1 DNA-binding domain contains a winged-helix domain most similar to the TFIIF Rap30 C-terminal winged-helix domain. Sfc1 DNA-binding domain recognizes single and double-stranded DNA by an unknown mechanism. Several features observed for A-box recognition by τA resemble the recognition of promoters by bacterial RNA polymerase, where σ factor unfolds double-stranded DNA and stabilizes the non-coding DNA strand in an open conformation. Such a function has also been proposed for TFIIF, suggesting that the observed structural similarity between Sfc1/Sfc7 and TFIIF Rap30/Rap74 might also reflect similar functions.

## INTRODUCTION

In most eukaryotes, three different RNA polymerases (Pol I, Pol II and Pol III) are responsible for the transcription of the genetic information. While Pol I synthesizes a single rRNA precursor, Pol II synthesizes all mRNAs and most non-coding RNAs, and Pol III synthesizes a subset of small RNAs, most notably tRNAs, 5S rRNA and U6 snRNA. Pol III mainly uses type 2 promoters to transcribe tRNA genes. Type 2 promoters contain two gene-internal DNA-binding elements named A- and B-box that are recognized by the general transcription factor IIIC (TFIIIC) (1). Sequence comparisons have defined 11 and 9 nt consensus sequences for A- and B-box, respectively (2). During Pol III transcription initiation, TFIIIC binds in a first step the B-box of tRNA genes with high affinity, which in a second step then allows TFIIIC binding to the A-box that is less stringently conserved and only weakly bound (3). TFIIIC binding is followed by the recruitment of TFIIIB and subsequently Pol III. As TFIIIC places TFIIIB at a defined distance upstream of the TFIIIC-binding site, and TFIIIB positioning determines the transcription start site, TFIIIC is involved in the selection of the transcription start site. The presence of TFIIIC in B-box-containing DNA regions has also been implicated in the spatial organization of the genomes from *Schizosaccharomyces pombe, Saccharomyces cerevisiae* and human, suggesting an additional role of TFIIIC as a chromosome-organizing clamp (4).

**Figure 1.**
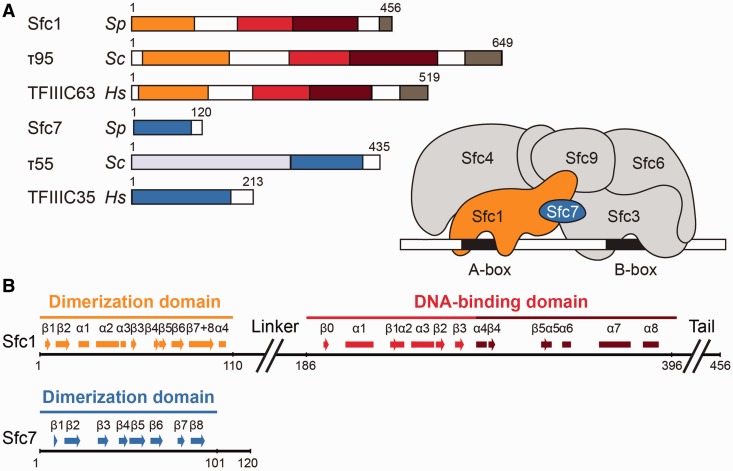
Overview of the TFIIIC complex. (**A**) Domain representation of *S. pombe* subunits Sfc1 and Sfc7 and comparison with their *S. cerevisiae* and *Homo sapiens* orthologues. In Sfc1, τ95 and TFIIIC63, the dimerization domain, winged-helix domain, WHI domain and the acidic tail are coloured in orange, red, dark red and grey, respectively. In Sfc7, τ55 and TFIIIC35, the dimerization domain is coloured in blue, and the *S. cerevisiae* τ55-specific histidine phosphatase domain in light blue. The right panel shows a schematic diagram of *S. pombe* TFIIIC bound to type 2 promoter DNA as present in tRNA genes. The Sfc1/Sfc7 heterodimer is depicted in orange and blue, while all other TFIIIC subunits are depicted in grey. (**B**) Domain structure of *S. pombe* Sfc1 and Sfc7. Secondary structure elements are indicated by bars (α-helices) and arrows (β-sheets). The colour code corresponds to (A).

TFIIIC is a six-subunit protein complex of ∼0.5 MDa mass consisting of two subcomplexes of similar size, τA and τB, which contact A-box and B-box promoter elements, respectively (5). The overall structural organization of the TFIIIC complex appears to be conserved from fungi to human, although conservation decreases when moving from the τA subcomplex close to the transcription start site towards the τB subcomplex further downstream of the start site (6). In the τA subcomplex, *S. cerevisiae* subunits τ131 (Tfc4), τ95 (Tfc1) and τ55 (Tfc7) correspond to *S. pombe* subunits Sfc4, Sfc1 and Sfc7, respectively, and to human subunits TFIIIC102, TFIIIC63 and TFIIIC35 (Figure 1A). In the τB subcomplex, *S. cerevisiae* subunits τ138 (Tfc3), τ91 (Tfc6) and τ60 (Tfc8) correspond to *S. pombe* subunits Sfc3, Sfc6 and Sfc9, respectively, and to human subunits TFIIIC220, TFIIIC110 and TFIIIC90. In subcomplex τA, subunit τ95 is the only subunit with DNA-binding properties, suggesting it is involved in A-box recognition (7). Subunits τ95 and τ55 have both been cross-linked over the A-box of tRNA genes approaching the DNA from opposite sides (8). In addition, they have both been found to form a subcomplex of unknown function that occurs independently of holoTFIIIC (9). The conserved C-terminal part of τ55 was shown to be sufficient for the τ95-τ55 interaction, whereas the non-conserved N-terminal histidine phosphatase domain is not required (10,11). In the τB subcomplex, subunits τ60 and τ91 both form WD40 propellers that are perpendicularly packed against each other (12). The third τB subunit, τ138 (Tfc3), directly contacts B-box DNA, possibly involving one or several winged-helix domains present in its N-terminal moiety.

The absence of detailed structural information on the τA complex of TFIIIC has impaired our current understanding of Pol III pre-initiation complex (PIC) formation. We therefore structurally characterized the *S. pombe* TFIIIC Sfc1/Sfc7 subcomplex and present crystal structures of two structural modules. The N-terminal moiety of Sfc1 (Sfc1-NT) and subunit Sfc7 heterodimerize through a triple β-barrel fold most similar to the dimerization domains of general transcription factor TFIIF Rap30/Rap74, whereas the C-terminal DNA-binding domain of Sfc1 (Sfc1-DBD) comprises a winged-helix domain most similar to the winged-helix domain present in TFIIF Rap30 that is packed against a second domain with novel fold. The Sfc1/Sfc7 heterodimer and TFIIF Rap30/74 therefore share similar N-terminal dimerization modules and C-terminal winged-helix domains but also similar overall architectures. Further biochemical characterization identifies Sfc1-DBD as the central DNA-binding domain (DBD) of A-box DNA that recognizes single- and double-stranded DNA (ssDNA and dsDNA) and is autoinhibited by a ∼60-residue C-terminal tail. Different DNA-binding modes of Sfc1-DBD to A-box DNA are principally possible. Comparison with primary σ factor domain 2 (σ70 in *Escherichia coli*) suggests one possible DNA-binding mechanism, where Sfc1-DBD might unfold double-stranded A-box DNA and stabilise one DNA strand in an open conformation.

## MATERIALS AND METHODS

### Production of recombinant proteins

*Schizosaccharomyces pombe* Sfc1 and Sfc7 constructs were cloned into the bacterial expression vectors pETM-11 and pCDF-MCN, respectively, thereby introducing a tobacco etch virus (TEV) protease-cleavable N-terminal His-tag into the Sfc1 protein. Full-length Sfc1 and Sfc7 and the Sfc1-NT/Sfc7 dimerization module were co-expressed overnight at 18°C in BL21 (DE3) RIPL *E. coli* cells. The Sfc1/Sfc7 complex was purified by Ni-NTA affinity chromatography, followed by TEV protease digestion overnight and a second Ni-NTA affinity chromatography step. Subsequently, the sample was diluted and loaded onto an anion exchange column (Mono Q HR10/10, GE Healthcare) followed by gel filtration (HiLoad 26/600 Superdex S200, GE Healthcare). Finally, the sample was concentrated, and limited proteolysis studies were performed (13). The Sfc1/Sfc7 dimerization module was purified using essentially the same procedure. The Sfc1-DBD (residues 186–396) was purified similarly to the Sfc1/Sfc7 interaction module using a cation exchange column (Q Sepharose High performance, GE Healthcare) instead of an anion exchange column, and without performing a gel filtration step. Further details for all purification procedures are given in the Supplemental Methods.

### Crystal structure determinations

The Sfc1_1-110(K48A,Q49A)_/Sfc7_1-101_ complex was crystallized by the hanging-drop vapour diffusion at 4°C by mixing 5 μl of protein solution, at a concentration of 8.5 g/l, with 5 μl of reservoir solution [0.1 M HEPES (pH 7), 1.4 M ammonium sulphate] and adding 0.5 μl of 1 M KCl. Crystals were harvested and subsequently derivatized by soaking them in mother liquor (without KCl) supplemented with 1 mM para-chloro-mercuribenzene sulfonate before back-soaking them in mother liquor (without KCl) containing 30% glycerol and flash-frozen in liquid nitrogen. A single-wavelength anomalous diffraction data set of the Hg-derivatized crystals was collected at European Synchrotron Radiation Facility (ESRF) beamline ID23-1. The data were processed using programs XDS (14) and Scala (15). SHELXD (16) as implemented in HKL2MAP (17) was used to solve the substructure, and final phasing was performed using program SHARP (18). Density modification was done using SOLOMON (19), followed by automatic building of an initial model with ARP/wARP (20). The model was modified and extended manually using COOT (21), refinement with phenix.refine (22) and model validation with MolProbity (23). Selenomethionine-containing Sfc1-DBD was prepared by inhibiting the methionine biosynthesis pathway as described (24). Crystals grew at 20°C using hanging-drop vapour diffusion. 1 μl of protein solution, at a concentration of 36 g/l, was mixed with 2 μl of reservoir solution containing 0.2 M MgCl_2_, 0.1 M Tris–HCl (pH 8.5) and 20% (w/v) PEG 3350. The crystals were harvested and cryo-protected with mother liquor supplemented with 20% glycerol, before being flash-frozen in liquid nitrogen. A multiple-wavelength anomalous diffraction (MAD) data set was collected at ESRF beamline BM30A, and data processing was performed using program XDS. The crystal structure was determined as described for the Sfc1_1-110(K48A,Q49A)_/Sfc7_1-101_ complex except that XSCALE was used instead of Scala, and the MAD method was used for experimental phasing. The SeMet data set collected at the peak of the absorption spectrum was used during the refinement.

### DNA-binding studies

Oligonucleotides containing the *S. pombe* A-box DNA site, the tRNA gene and unspecific DNA sequences, used in the DNA-binding assays, are given in the Supplemental Methods. High pressure liquid chromatography-purified oligonucleotides were labelled with ^32^P by T4 polynucleotide kinase and gel-purified on denaturing 15% urea–PAGE. Radioactively labelled ^32^P oligonucleotides were first annealed by heating in water at 95°C for 1 min, cooled down to 25°C and incubated in buffer D [20 mM Tris–HCl (pH 7.5), 200 mM KCl, 2 mM MgCl_2,_ 10 mM DTT]. For the electrophoretic mobility shift assays (EMSA) ^32^P-labelled DNA was incubated with Sfc1-DBD constructs in buffer D. After incubation at 25°C, the samples were loaded on a native 6% acrylamide gel and run at 160 V in Tris–glycine buffer. For the competition experiments, ^32^P-labelled A-box was first bound to 1 μM Sfc1-DBD. Then, increasing concentrations of Sfc1_434-456_ peptide and control peptides (1.10^−6^ to 3.10^−4 ^M) were added to challenge the complex. After incubation at 25°C for 30 min, the reactions were loaded on a native 6% acrylamide gel. A blank reaction and a control without peptide were also carried out. After migration, the native gel was dried and autoradiographed for 2 h with X-ray film (Biomax, MR-film, Kodak).

For filter-binding assays, proteins were serially diluted (from 100 μM to 0.1 nM) in a final volume of 180 μl of buffer D, followed by addition of 20 μl of renatured ^32^P-labelled DNA (∼5000 cpm). After incubation at 25°C, the reactions were filtered through a nitrocellulose filter and the radioactivity counted. For each series, a blank reaction and the total DNA input were determined. Because the amount of labelled DNA is negligible, an apparent dissociation coefficient (*K*_D_) can be estimated from the concentration value at 50% DNA retention (25).

### Isothermal titration calorimetry

Isothermal titration calorimetry (ITC) measurements were performed at 25°C using a VP-ITC Microcal calorimeter (Microcal, Northhampton, USA). Protein was dialysed overnight against ITC buffer [50 mM HEPES (pH 7.5), 150 mM NaCl, 2 mM β-mercaptoethanol]. The lyophilized peptide was re-suspended in ITC buffer. The titration experiments consisted of injecting 10 µl of a 200 µM peptide solution into 2 ml of a 10 µM protein solution at time intervals of around 5 min.

## RESULTS

### Sfc1/Sfc7 heterodimer consists of two separate modules

The *S. pombe* Sfc1/Sfc7 heterodimer was co-expressed in *E. coli* and purified to homogeneity. Limited proteolysis to investigate the domain organization of the Sfc1/Sfc7 heterodimer resulted in three different species: Sfc1 (M_r_ = 52.7 kDa) was rapidly cleaved into two protease resistant fragments, one similar in size to Sfc7 (M_r_ = 13.3 kDa) and one ∼25 kDa in size, whereas Sfc7 was resistant to proteolysis. Sfc7 and the smaller Sfc1 fragment remained stably associated during ion-exchange chromatography, unlike the second larger Sfc1 fragment. Electrospray mass spectrometry in combination with peptide mass fingerprinting allowed the assignment of the smaller Sfc1 fragment interacting with Sfc7 to residues 1–107 (Sfc1-NT) and the larger Sfc1 fragment to residues 186–396 (corresponding to Sfc1-DBD). In contrast, the residues that connect Sfc1-NT with Sfc1-DBD and the C-terminal extension following Sfc1-DBD are readily degraded (Figure 1B). The Sfc1-NT/Sfc7 heterodimer did not interact with the larger fragment during gel filtration, indicating that the Sfc1/Sfc7 heterodimer consists of two separate globular modules connected by a protease sensitive linker.

### Dimerization modules of Sfc1/Sfc7 and TFIIF Rap30/Rap74 are similar

**Figure 2.**
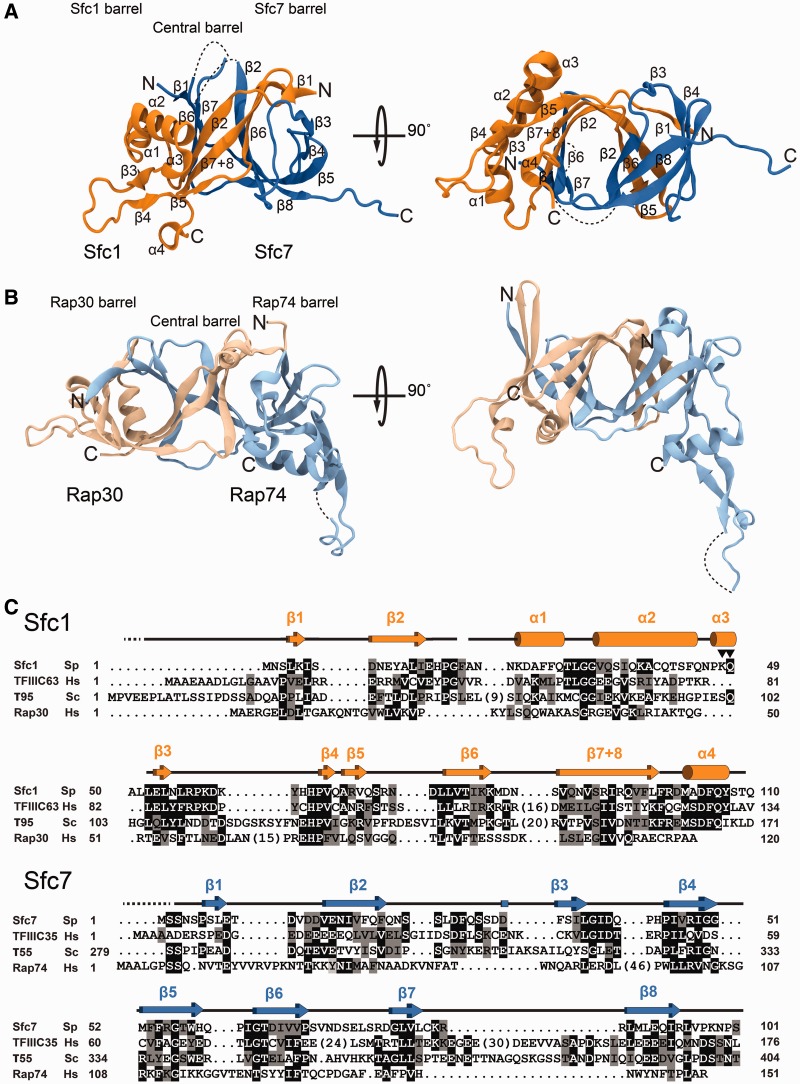
Structure of the Sfc1/Sfc7 dimerization domains. (**A**) Two orthogonal views of the Sfc1-NT/Sfc7_1-101_ dimerization domains. Sfc1-NT is depicted in orange, whereas Sfc7_1-101_ is depicted in blue. Secondary structure elements and N- and C-termini are labelled. Disordered loops are represented by dashed lines in (A) and (B). (**B**) Two orthogonal views of the TFIIF Rap30/Rap74 dimerization domains. Rap30 is depicted in light orange, and Rap74 is depicted in light blue. The orientation is the same as for the Sfc1/Sfc7 dimerization domains in (A). (**C**) Sequence alignment of the N-terminal moieties of *S. pombe* Sfc1 *(Sp)*, human TFIIIC63 (*Hs*), *S. cerevisiae* τ95 (*Sc*) and human TFIIF Rap30 *(Hs)*, and of *S. pombe* Sfc7 (*Sp*), human TFIIIC35 (*Hs*), the C-terminal part of *S. cerevisiae* τ55 (*Sc*) and human TFIIF Rap74 *(Hs)*. The α-helices and β-sheets are depicted as cylinders and arrows, respectively. Sequence conservation of identical and similar residues is represented by dark and light grey shading, respectively. Arrowheads indicate Sfc1 K48 and Q49 residues that were mutated to alanine to reduce surface entropy.

Well-diffracting crystals were obtained for a Sfc1_1-110(K48A,Q49A)_/Sfc7_1-101_ construct, where two non-conserved Sfc1 residues (K48 and Q49) were mutated into alanine following the suggestion of the surface entropy reduction prediction (SERp) server (26). The structure was solved at 2.4 Å by single-wavelength anomalous diffraction using para-chloro-mercuribenzene sulfonate as heavy-atom derivate (Table 1). The Sfc1-NT/Sfc7 dimerization module consists of three interwoven β-barrels containing 15 β-strands and four α-helices (Figure 2A and C). The interwoven structure suggests that the subunits fold co-operatively and need to be simultaneously expressed. Indeed, in this study, Sfc1_1-110(K48A,Q49A)_ and Sfc7_1-101_ (as well as full-length Sfc1 and Sfc7) were co-expressed in *E. coli* because it had been observed that the expression of isolated full-length Sfc1 resulted in insoluble protein. The crystal structure also confirmed that the mutated residues K48 and Q49 lay at the surface of the Sfc1 dimerization domain and do not interfere with Sfc1–Sfc7 heterodimerization. According to program Dali (27), the structure shares the highest similarity with the TFIIF Rap30/Rap74 dimerization domains (28). Sfc1 and Sfc7 resemble Rap30 and Rap74, respectively (*Z*-score = 9.3, rmsd_154CA_ = 4.7 Å) (Figure 2B). Because of the pseudo 2-fold symmetry of the triple-barrel fold, Sfc1 and Sfc7 also superimpose with Rap74 and Rap30, respectively, although with lower *Z-*score. Despite the readily apparent structural similarity between Sfc1 and Rap30 and Sfc7 and Rap74, Sfc1 helices α1 to α3 and β-strands β3 and β4 replace Rap30 strands β3 and β4 (approximately residues 20–68), whereas the ‘arm domain’ of Rap74 is absent in Sfc7. The Sfc1-NT/Sfc7 dimerization domain can also be superimposed with the dimerization modules of Pol I subunits A49/A34.5 (*Z*-score = 7.7, rmsd_142CA_ = 4.2 Å) and mouse RNase H2B/H2C (*Z*-score = 7.8, rmsd_134CA_ = 3.7 Å).

**Table 1.** Crystallographic statistics for the Sfc1-NT/Sfc7 complex

	Sfc1-NT/Sfc7 complex (Hg-derivative)
Data collection	
Space group	P6_3_22
Cell dimensions	a, b = 85.3
a, b, c (Å)	c = 154.1
Wavelength (Å)	1.00685
Resolution (Å)	42.6-2.40 (2.53–2.40)
R_sym_ (%)	10.7 (88.8)
<I/σ(I)>	14.5 (2.1)
Completeness (%)	99.9 (99.6)
Multiplicity	12.1 (9.4)
Refinement	
Resolution (Å)	33.32.40
No. reflections	24,503
* R_work_*/*R_free_* (%)	18.1/22.4
No. atoms	
Protein	1534
Other	3
Water	42
B-factor (Å^2^)	
Protein	56.1
Other	92
Water	49.7
R.m.s. deviations	
Bond lengths (Å)	0.011
Bond angles (°)	1.318

### Sfc1-DBD and TFIIF Rap30 contain similar winged-helix domains

**Figure 3.**
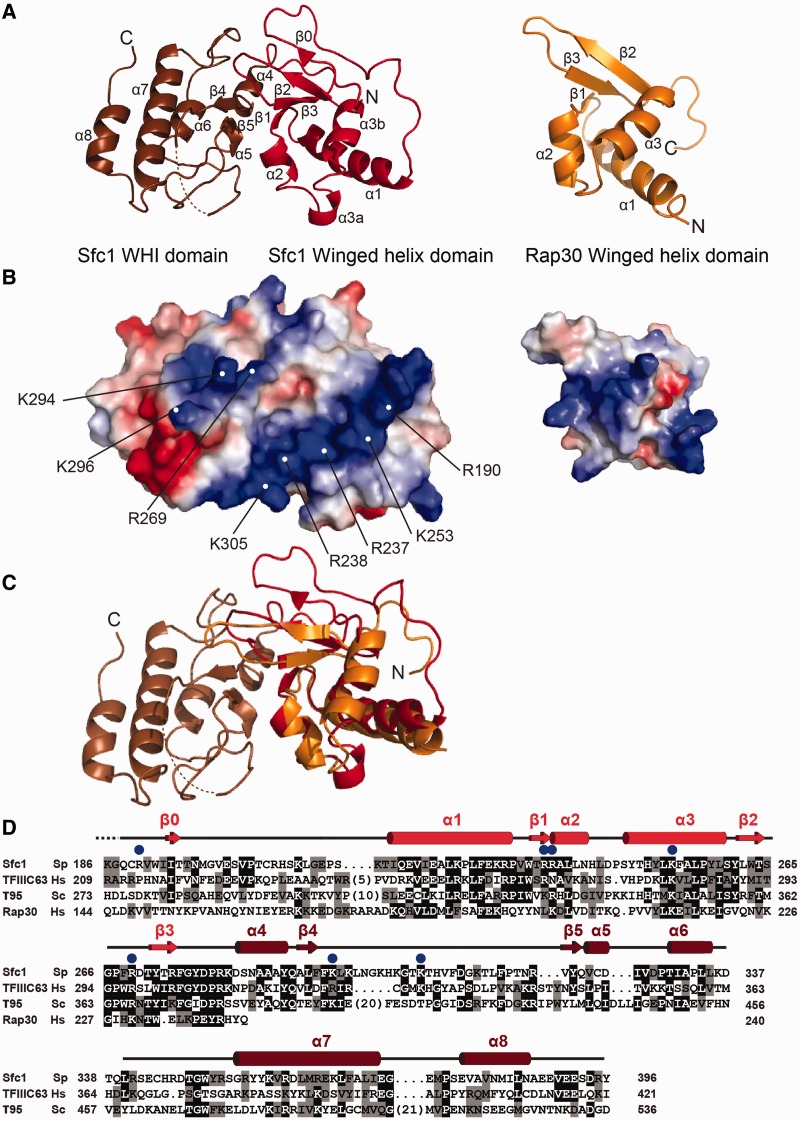
Structure of Sfc1-DBD. (**A**) Ribbon representation of Sfc1-DBD. The winged-helix domain is depicted in bright red, and the WHI domain is depicted in dark red. The right panel shows the winged-helix domain of TFIIF Rap30 in the same orientation. (**B**) Electrostatic surface charge representation of Sfc1-DBD. Residues that contribute forming the small and large basic patches are labelled. The right panel shows the electrostatic surface charge of the TFIIF Rap30 winged-helix domain. (**C**) Superposition of the winged-helix domains of Sfc1-DBD and Rap30. (**D**) Sequence alignment of *S. pombe* Sfc1-DBD (*Sp*) with corresponding regions of *H. sapiens* TFIIIC63 (*Hs*), *S. cerevisiae* τ95 *(Sc*) and human TFIIF Rap30 *(Hs)*. Secondary structure elements present in Sfc1-DBD are depicted as cylinders (α-helices) and arrows (ß-sheets). Blue dots indicate conserved basic Sfc1-DBD residues mutated for DNA-binding studies.

The crystal structure of the C-terminal domain of Sfc1 (Sfc1-DBD, residues 186–396) was solved at 1.45 Å resolution by MAD using selenomethionine-substituted protein (Table 2). The Sfc1-DBD structure forms a compact domain that can be further divided into two subdomains (Figure 3A and D). The first domain (residues 186–283) adopts a winged-helix domain fold with canonical α1 -β1-α2-α3-β2-β3 topology. One additional strand (β0) pairs strand β2 of the winged-helix domain in an anti-parallel manner. The third helix α3 is not continuous, but split into two short helices α3a and α3b. The second domain (residues 284–396) consists of five α-helices and two β-strands and adopts a novel fold with no significant similarity to any known structure. Subsequently, we will refer to this domain as winged-helix-interacting (WHI) domain. Structural comparison using program DALI identifies the winged-helix domain of Rap30 (29) as the structure with the closest similarity to the winged-helix domain of Sfc1 (*Z*-score = 6.1, rmsd_61CA_ = 2.7 Å) among the many winged-helix domains present in the PDB database (Figure 3C). Sfc1 therefore resembles TFIIF Rap30 in its N-terminal dimerization domain and in its C-terminal DBD.

**Table 2.** Crystallographic statistics for Sfc1-DBD

	SeMet Sfc1-DBD		
	Peak	Inflection point	Remote
Data collection			
Space group		P2_1_2_1_2_1_	
Cell dimensions			
a, b, c (Å)		35.4, 67.6, 88.1	
Wavelength (Å)	0.980062	0.980199	0.978558
Resolution (Å)	50.0-1.45 (1.49-1.45)	50.0-1.45 (1.49-1.45)	50.0-1.45 (1.49-1.45)
R_sym_ (%)	4.4 (28.9)	4.8 (30.0)	4.3 (28.7)
<I/σ(I)>	19.23 (2.85)	19.17 (2.71)	20.89 (2.81)
Completeness (%)	95.8 (74.0)	96.5 (75.9)	96.5 (76.2)
Multiplicity	3.01 (1.74)	3.28 (1.87)	3.27 (1.87)
Refinement			
Resolution (Å)	44.0–1.45		
No. reflections	69,339		
* R_work_*/*R_free_* (%)	15.2/17.0		
No. atoms			
Protein	1720		
Water	258		
B-factor (Å^2^)			
Protein	17.7		
Water	29.4		
R.m.s. deviations			
Bond lengths (Å)	0.012		
Bond angles (°)	1.339		

### Sfc1-DBD binds DNA with an extended basic region

Inspection of the Sfc1-DBD electrostatic surface potential identifies an extended basic region on one side of the Sfc1-DBD that could interact with DNA (Figure 3B), whereas the opposite side appears rather uncharged. The basic interaction surface of Sfc1-DBD shows two separate positively charged patches. One continuous larger patch runs diagonally across the surface of Sfc1-DBD and predominantly comprises basic residues of helices α2 and α3 of the winged-helix domain. The second smaller patch comprises residue R269 of the winged-helix domain and residues K294 and K296 of the WHI domain (Figure 3B). Almost all residues forming both basic patches are conserved between TFIIIC Sfc1 orthologues (Figure 3D).

**Figure 4.**
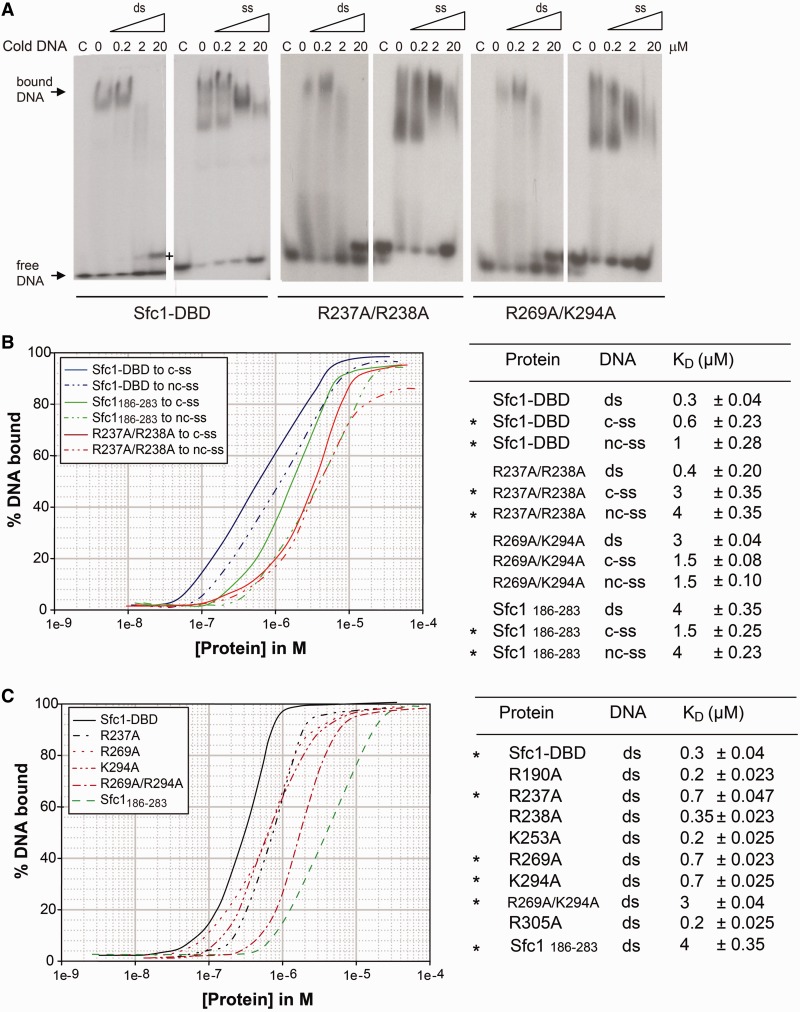
DNA binding of Sfc1-DBD to ssDNA and dsDNA. (**A**) EMSA of Sfc1-DBD and Sfc1-DBD mutant proteins bound to 25meric ssDNA (panel 1) and dsDNA (panel 2). Arrows indicate Sfc1-DBD-DNA complexes. Increasing amounts of non-radioactive (cold) dsDNA or ssDNA were added to compete for binding. The amounts of single-stranded or double-stranded competitor DNA are depicted above the lane in μM. The same experiments using mutant proteins Sfc1-DBD R237A/R238A and R269A/K294A that change the large and small basic patches, respectively, are depicted in panels 3/4 and 5/6. In panel 1, plus indicates dsDNA formed through strand-exchange with radioactive ssDNA. (**B**) Filter binding assays of Sfc1-DBD, Sfc1_186-283_ lacking the WHI domain, Sfc1-DBD R237A/R238A and Sfc1-DBD R269A/K294A mutant proteins bound to dsDNA (ds), non-coding ssDNA (nc-ss) and coding ssDNA (c-ss). The resulting K_D_ values are listed in the right table. Asterisks in (B) and (C) indicate those experiments where the corresponding experimental data are depicted in the left panel. (**C**) Filter binding assays of Sfc1-DBD and Sfc1-DBD mutant proteins bound to a 25 bp, double-stranded A-box DNA site. As in (B), resulting K_D_ values are listed in the right table.

In a next step, we tested binding of Sfc1-DBD to dsDNA and ssDNA DNA oligonucleotides using electrophoretic mobility shift assays (EMSA) (Figure 4A) and filter binding experiments (Figure 4B). Sfc1-DBD binds 25 bp dsDNA as well as ssDNA. Consistent with a minor contribution of A-box binding to the overall binding affinity of TFIIIC (30), competition experiments and filter binding assays showed only μmolar binding affinities of Sfc1-DBD for dsDNA and ssDNA (Figure 4A and B). The competition experiments also suggested that dsDNA and ssDNA use overlapping binding sites as dsDNA is able to replace ssDNA and *vice versa*. Binding to dsDNA and ssDNA was also tested for Sfc1-DBD R237A/R238A and R269A/K294A mutant proteins that modify the large and small positively charged patches, respectively. Both mutant proteins are still able to bind ssDNA and dsDNA with similar reduced affinities (Figure 4A and B), suggesting that both patches are involved in ssDNA and dsDNA binding and are required for the full DNA-binding capacity of Sfc1-DBD. Accordingly, the winged-helix domain (residues 186–283) lacking the WHI domain still binds dsDNA and ssDNA, although with ∼10-fold reduced affinity compared to entire Sfc1-DBD (Figure 4B and C). Also consistent with the results of endogenous TFIIIC binding to the A-site (29), Sfc1-DBD possesses little sequence specificity for A-box containing DNA oligonucleotides, as it binds with similar affinity to B-box dsDNA (data not shown) and to coding and non-coding ssDNA (Figure 4B). To probe the contribution of individual Sfc1-DBD residues more quantitatively, we tested the binding affinities for dsDNA of a wide range of structure-based site-directed mutants (Figure 4C). Several point mutants had an inhibitory effect on dsDNA binding decreasing the affinities by more than 2-fold. These included mutants R237A in helix α2, R269A in the ‘wing’ connecting strands β1 and β2, single mutant K294A in the loop connecting strands β4 and β5 of the WHI domain and double mutant R269A/K294A (Figure 4C). The observed low-affinity DNA binding with little sequence specificity of Sfc1-DBD well agrees with the DNA-binding characteristics observed for endogenous TFIIIC purified from *S. cerevisiae*. Total deletion of the A-box only decreased the equilibrium constant for TFIIIC by a factor 2- to 5-fold (30), whereas ssDNA can interfere with TFIIIC binding to the A-box and higher temperature increases the stability of the TFIIIC-DNA complex (31) (see ‘Discussion’ section later in the text).

### The acidic tails of Sfc1 inhibits its DNA binding activity

**Figure 5.**
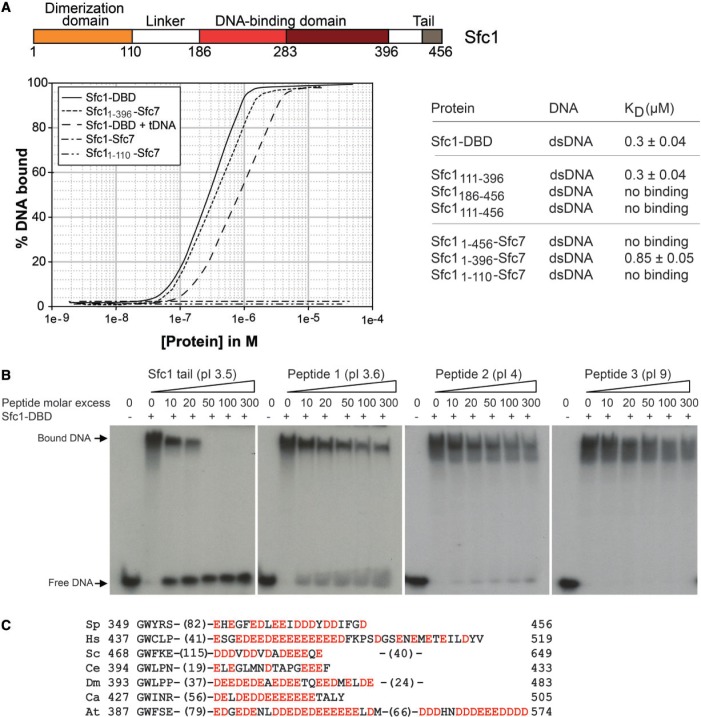
Auto-inhibition of the DNA binding activity of the Sfc1/Sfc7 heterodimer by its C-terminal tail. (**A**) Filter binding assays of different Sfc1 and Sfc1/Scf7 heterodimer constructs to a 25 bp double-stranded A-box DNA site. The domain structure of Sfc1 is given as reference. (**B**) EMSA of Sfc1-DBD bound to radioactively labelled DNA with the 22mer acidic Sfc1 tail peptide (Sfc1_435-456_) added in increasing amounts to compete for DNA-binding (left panel). Control peptides 1–3 compete less efficiently for DNA binding (three right panels). The molar excess of tail peptide to protein is indicated. For each peptide pI value, length and sequence is given: Sfc1 tail, pI 3.5, 22mer: QEHEGFEDLEEIDDDYDDIFGD. Peptide 1, pI 3.6, 28mer: HLIQDEEYDDEDVPHDLQLSEDEYNSER. Peptide 2, pI 4, 31mer: NSKRLEEDNDEEQSHHKKTKQAVSYDEPDED. Peptide 3, pI 9: 23mer: QLPPPPKLSMVGFPLLYKYKANP. (**C**) Alignment of acidic tail residues from Sfc1 *S. pombe* (Sp) with orthologues from human (Hs), *S. cerevisiae* (Sc), *C. elegans* (Ce), *Drosophila melanogaster* (Dm), *Candida albicans* (Ca), *Arabidopsis thaliana* (At)*.* Acidic residues are depicted in red.

We observed a stretch of acidic residues in the C-terminal extension following Sfc1-DBD that is conserved between different Sfc1-orthologs. To further explore the function of these acidic residues, we compared the DNA-binding affinity of Sfc1-DBD and construct Sfc1_111-396_ that contains 75 additional N-terminal residues with longer constructs Sfc1_186-456_ and Sfc1_111-456_ comprising the C-terminal 60-residue extension including the conserved acidic residues. Interestingly, the constructs including this extension did no longer bind dsDNA (Figure 5A), suggesting an auto-inhibitory activity in this region. The Sfc1_1-396_/Sfc7 heterodimer lacking the C-terminal tail also binds dsDNA, whereas the Sfc1/Sfc7 heterodimer that includes the C-terminal tail did not bind DNA, demonstrating that the C-terminal acidic tail of Sfc1 also inhibits DNA binding in the context of the Sfc1/Sfc7 heterodimer. Subsequently, we tested whether the most conserved region of acidic residues in the C-terminal tail might be sufficient for this inhibition. Indeed, a synthetic peptide comprising only 22 C-terminal residues of Sfc1 (Sfc1_435-456_) was able to bind to Sfc1-DBD with an affinity of 3.0 ± 0.8 μM as shown by isothermal calorimetry (data not shown). The same peptide was also able to disrupt binding of Sfc1-DBD to DNA, when it was titrated in during an EMSA (Figure 5B), suggesting that Sfc1 residues 435–456 are sufficient for the autoinhibition of the DNA-binding activity of Sfc1-DBD. The binding of the acidic tail to Sfc1-DBD appears to be specific because other peptides of similar lengths and pI values, but with unrelated amino acid sequences, are less efficient in disrupting DNA binding (Figure 5B). Cryptic DBDs have been also described in TFIIF Rap30 (32) and TFIIIB subunit Brf1 (33). Autoinhibitory regions generally increase specificity of DNA recognition, as they only allow DNA binding under well-defined conditions, thereby preventing non-specific binding. Although the acidic C-terminal tail is conserved between different species (Figure 5C), its functional importance might vary between species. In *S. cerevisiae*, the corresponding C-terminal residues in subunit τ95 are not required for viability at least under normal growth conditions (34).

## DISCUSSION

### Role of the Sfc1-Sfc7 heterodimer within TFIIIC

Here, we provide first insight into the structural organization of two subunits of the TFIIIC τA subcomplex. Our results identified the *S. pombe* Sfc1-Sfc7 heterodimer as stable subcomplex within TFIIIC that comprises a Sfc1-Sfc7 triple-β-barrel dimerization module and a DBD in the C-terminal moiety of Sfc1. In *S. cerevisiae*, the corresponding τ95-τ55 complex has been also described as an independent heterodimer (9). Additional interactions with other TFIIIC subunits occur between the Sfc1 linker region that connects the Sfc1 dimerization domain and its C-terminal DBD and τA subunit Sfc4 (data not shown). At present, it is unknown whether the Sfc1-Sfc7 heterodimer also interacts with any of the τB subunits. However, *S. cerevisiae* τ95-E447K mutant (corresponding to Sfc1-DBD residue D328) considerably destabilizes the entire TFIIIC-DNA complex (35). Because binding of τA to the A-box contributes little to the overall DNA-binding affinity of TFIIIC (see earlier in the text), this suggested that τ95 might also interact with τB subunits thereby indirectly influencing the binding to these subunits to the B-box (35). Sfc1-DBD interacts with ssDNA and dsDNA with little sequence specificity. Binding of ssDNA and dsDNA involves overlapping binding sites as all site-directed mutants similarly affect ssDNA and dsDNA binding. The binding affinities of Sfc1-DBD and the Sfc1-Sfc7 heterodimer to a 25 bp dsDNA target site and of the entire τA complex to a longer DNA target site (data not shown) are similar. This suggests that the Sfc1-DBD contains most of the important residues required for A-box binding and that Sfc1-DBD is the complete DBD of the τA subcomplex.

### DNA recognition by Sfc1-DBD

**Figure 6.**
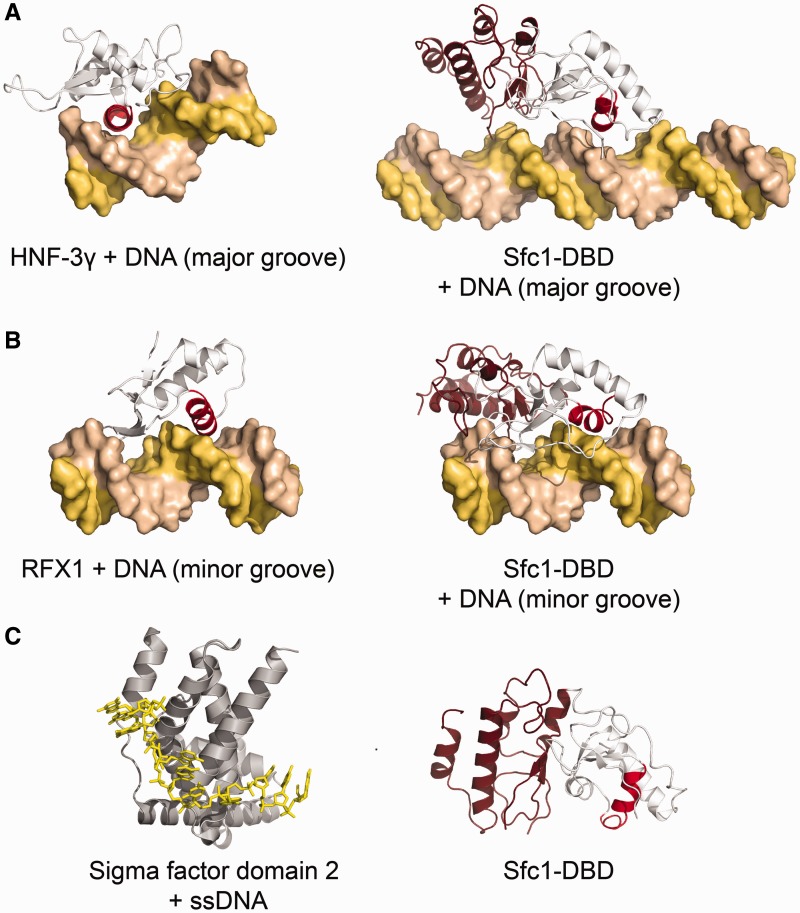
DNA recognition by Sfc1-DBD. (**A**) dsDNA recognition by HNF-3γ (PDB ID: 1VTN) with the recognition helix coloured in red contacting the major groove of the DNA (left panel). Sfc1-DBD is depicted in the same orientation contacting ideal B-form DNA in the major groove through its recognition helix coloured in red. (**B**) dsDNA recognition by RFX1 (PDB ID: 1DP7) with the recognition helix contacting the minor groove of the DNA (left panel). Sfc1-DBD is depicted in the same orientation contacting ideal B-form DNA in the minor groove. (**C**) ssDNA recognition by σ factor domain 2 (PDB ID: 3UGO). Sfc1-DBD is presented in the right panel as reference.

Our results show that Sfc1-DBD is able to recognize ssDNA and dsDNA, although the way the Sfc1-DBD recognizes A-box DNA still requires further analysis. Superimposing Sfc1-DBD onto the crystal structure of a typical winged-helix domain-DNA complex (HNF-3γ-DNA) bound to dsDNA, where the recognition helix H3 protrudes into the major groove, shows that DNA recognition by Sfc1-DBD considerably differs from canonical B-form DNA-recognition by winged-helix domains (36). Although the Sfc1-DBD recognition helix approximately fits into the major groove of B-form DNA (Figure 6A), adjacent conserved residues immediately following strand β4 of the WHI domain (residues FKLK) would clash with the DNA backbone. To avoid clashes and to maximize interaction between Sfc1-DBD and dsDNA, the DNA needs to bend in such a way that the recognition helix α3 could still bind into the major groove, whereas residues FKLK could reach into the minor groove of the DNA. A second alternative dsDNA-binding mode is also possible, where the recognition helix contacts the minor groove as observed in the RFX1-DNA complex (36), whereas residues of the WHI domain would contact the major groove (Figure 6B).

The crystal structure of the bacterial σ factor domain 2 bound to a single-stranded −10 element sequence (37) suggests yet a third alternative DNA-binding mode for the binding of Sfc1-DBD to A-box DNA. In the σ factor domain 2/DNA complex, the dsDNA promoter is opened, the non-template strand is extruded, and DNA bases of the single strand are recognized by intricate hydrophobic and polar interactions resulting in the sequence-specific readout of the −10 element sequence (Figure 6C). The binding characteristics of Sfc1-DBD and the Sfc1-Sfc7 heterodimer described here and of TFIIIC purified from *S. cerevisiae* (31) are consistent with a similar DNA-binding mode. Stillman *et al.* observed that τA binding to the A-box contributes little to the TFIIIC binding affinity, whereas τB binding to the B-box provides the major contribution. Furthermore, τA binding to the A-box appears to be temperature dependent, not sequence-specific and can be inhibited by ssDNA. These observations had led to the suggestion that the A-box undergoes a closed to open transition during τA binding similar as observed during promoter opening by bacterial DNA-dependent RNA polymerases (31). Interestingly, a similar role in promoter opening has been also proposed for TFIIF Rap30 that also shares some sequence similarity with bacterial σ factor domain 4 (32).

### Similarities between TFIIIC Sfc1/Sfc7 and TFIIF Rap30/Rap74

**Figure 7.**
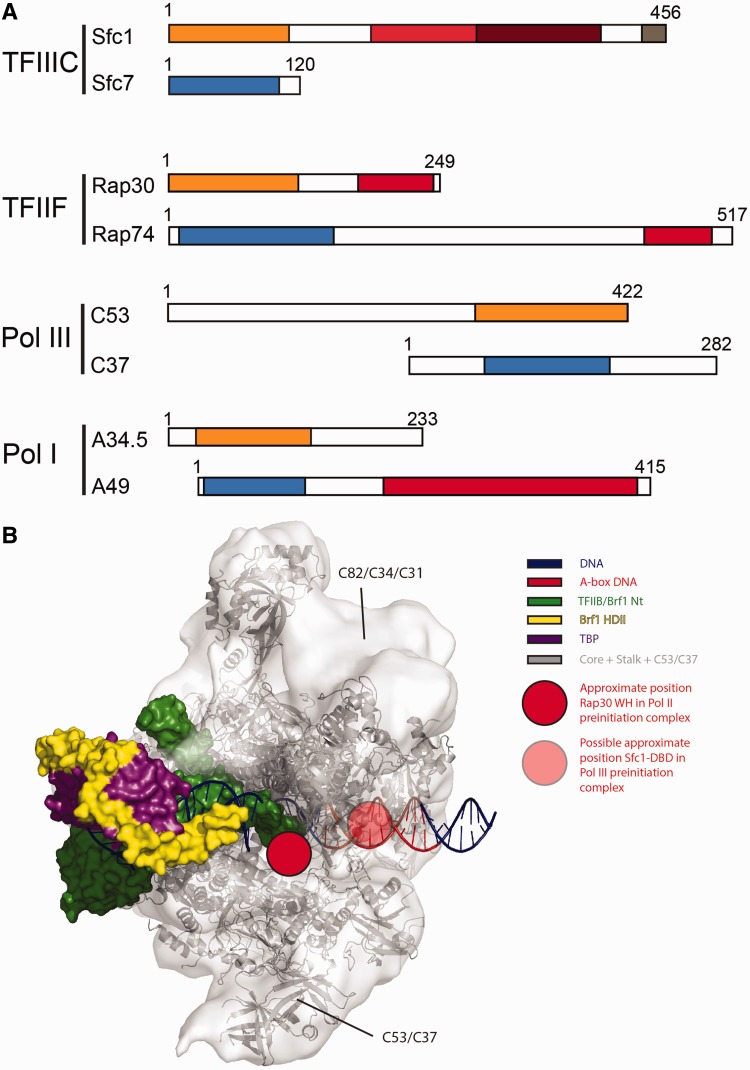
TFIIF-like role of the Sfc1/Sfc7 heterodimer in transcription. (**A**) Overall domain organization of TFIIIC Sfc1/Sfc7, TFIIF Rap30/Rap74, Pol III C53/C37 and Pol I A34.5/A49. Dimerization domains related to TFIIF Rap30 and Rap74 are depicted in orange and blue, respectively. The winged-helix domains in TFIIF Rap30, Rap74, Sfc1-DBD and the tandem winged-helix domain in A49 are depicted in bright red, the Sfc1-WHI domain in Sfc1 dark red, and the Sfc1 acidic tail in dark grey. For TFIIIC, the *S. pombe* orthologues, for TFIIF, the human orthologues and for Pol I and III, the *S. cerevisiae* orthologues are depicted. (**B**) Model of the Pol III open preinitiation complex. The open PIC complex was prepared by fitting the elongation complex of the Pol II structure (PDB ID: 1I6H) and the homology model of the C53/C37 dimerization domains into the Pol III EM density map (EMDB code 1802). Yeast TFIIB residues 22–211 were obtained from the initially transcribing Pol II complex in complex with TFIIB (PDB ID: 4BBS) after superimposing the Pol II Rpb2 subunits of this structure and the elongation complex. Crystal structures of Pol II-TFIIB (PDB ID: 3K7A), TFIIB-TBP-DNA (PDB ID: 1VOL) and the Brf1 homology region II in complex with TBP and DNA (PDB ID: 1NGM) were used to complete the open PIC. DNA was extended using B-form DNA with the position of the A-box (basepair +8 to +19) indicated in red. Red and transparent red dots indicate the positions of Rap30 winged-helix domain (54) and of Sfc1-DBD, respectively. The colour code is given in the legend at the right.

Unexpectedly, the TFIIIC Sfc1/Sfc7 heterodimer resembles the Pol II-specific general transcription factor TFIIF. In mammals and *Drosophila*, TFIIF consists of subunits Rap30 and Rap74, whereas yeast TFIIF comprises subunits Tfg2 and Tfg1 and a non-essential third subunit (38). TFIIF is required for RNA polymerase II PIC assembly (39) and interacts directly with DNA and other PIC components (40–42). TFIIF is also required for open complex formation (43) and transcription start site selection (44,45) and has been shown to function *in vitro* during transcriptional elongation (43). The overall domain architecture of TFIIF Rap30/Rap74 and TFIIIC Sfc1/Sfc7 is similar (Figure 7A). Both heterodimers use N-terminal triple β-barrel domains as dimerization modules (28) and TFIIF Rap30/Rap74 and TFIIIC Sfc1 (but not Sfc7) contain winged-helix domains as cryptic DBDs (29,46). The N-terminal region of Sfc1 involved in forming the triple β-barrel domain is most similar to Rap30, whereas the Sfc1 winged-helix domain is most similar to the winged-helix domain of Rap30 (among all winged-helix domains present in the PDB), suggesting a possible common origin of Sfc1 and TFIIF Rap30.

Distant homologies have been also identified between Pol II-specific general transcription factor TFIIF Rap30/Rap74, Pol I subunits A34.5/A49 and Pol III subunits C53/C37, but also between the Pol II-specific general transcription factor TFIIE and Pol III subunits C34 and C82 [reviewed in (47)]. This has led to the suggestion that Pol I and Pol III permanently recruited general transcription factors while they are dissociable in the Pol II enzyme (48). Pol I subunits A34.5/A49 and Pol III subunits C53/C37 are similarly positioned on the RNA polymerase core and are thought to be the functional equivalent to TFIIF Rap30/Rap74 (49–52). Although detailed structural information of the entire C37/C53 heterodimer or parts of it are lacking, comparison of the overall domain organization of TFIIIC Sfc1/Sfc7 and Pol III C53/C37 heterodimers suggests that the Sfc1/Sfc7 heterodimer even closer resembles TFIIF Rap30/Rap74 than Pol III C53/C37. Sfc1 and TFIIF Rap30 share a similar overall domain organization where the dimerization domain is followed by a winged-helix domain. In contrast, in C53 the dimerization domain is located at the C-terminus (Figure 7A). Furthermore, we could not find any predicted winged-helix domains for *S. cerevisiae* or *S. pombe* C53 and C37, nor for human C53, using homology modelling (53). In contrast, the human homologue of C37 (which has been described to be more homologous to Rap74 and is considerably larger than its yeast homologues) potentially contains one or two winged-helix domains. Recent cryo-electron microscopy reconstructions located the C-terminal Rap30 winged-helix domain in the human Pol II transcription initiation complex (54). The corresponding position in Pol III is indicated and lies in close proximity to the A-box DNA site recognized by Sfc1-DBD in a model of an initial open Pol III complex (Figure 7B). The C53/C37 subcomplex has been initially implicated in transcription termination (55), but it is also involved in transcription initiation and promoter opening (56). The C53/C37 dimerization module (similar as the corresponding modules in TFIIF Rap30/Rap74 and in A34.5/A49) occupies a position at one side of the Pol III DNA-binding cleft (Figure 7B). Photo-cross-linking experiments demonstrated that the N-terminal extension of C53 reaches across the DNA-binding cleft and contacts Pol III-specific subunits C34 and C82, the Pol III stalk and subunit TFIIIC τ131, whereas the C-terminal extension of subunit C37 contacts the active center of Pol III and presumably also the DNA transcription bubble (57). Given their close spatial proximity, Sfc1/Sfc7 could assist C53/C37 during Pol III transcription. Accordingly, the C53/C37 heterodimer would initiate promoter opening, whereas the Sfc1/Sfc7 heterodimer would bind to the internal A-box promoter DNA, subsequently Sfc1-DBD would extrude and stabilize the non-coding DNA strand similar as observed in the σ factor domain 2/ssDNA complex. Open complex stabilization by the Sfc1/Sfc7 heterodimer would allow Pol III to transcribe across the internal A-box promoter site of tRNA genes, while maintaining contacts with TFIIIC, as it has been postulated as requirement for ‘facilitated re-initiation’ during Pol III transcription. Indeed, the high rate of transcription at Pol III genes, together with the short length of these genes, suggests the possibility that a large part of Pol III tRNA genes could be single-stranded during active transcription. It might be energetically more favourable to keep the gene-internal promoter open rather than to repeatedly open and close the DNA. The fact that the ssDNA-binding factor Sub1 plays an important role in Pol III transcription further supports this hypothesis (58,59).

## ACCESSION NUMBERS

Coordinates and structure factors have been deposited in the Protein Data Bank with accession numbers 4BJJ and 4BJI for Sfc1_1-110(K48A,Q49A)_/Sfc7_1-101_ and Sfc1-DBD, respectively.

## SUPPLEMENTARY DATA

Supplementary Data are available at NAR Online.

Supplementary Data
