# Supplementary material for: RNA polymerase III-specific general transcription factor IIIC contains a heterodimer resembling TFIIF Rap30/Rap74
Source: Nucleic Acids Res. 2013 Aug 5;41(19):9183–96. doi: 10.1093/nar/gkt664 (PMC3799434; doi:10.1093/nar/gkt664)
Supplement: Supplementary Data [file supp_41_19_9183__index.html]

RNA polymerase III-specific general transcription factor IIIC contains a heterodimer resembling TFIIF Rap30/Rap74 — RNA polymerase III-specific general transcription factor IIIC contains a heterodimer resembling TFIIF Rap30/Rap74 — Supplementary Data 

# RNA polymerase III-specific general transcription factor IIIC contains a heterodimer resembling TFIIF Rap30/Rap74

## 

files

**Files in this Data Supplement:**

- Supplementary Data - pdf file
