# Supplementary material for: RNA polymerase III-specific general transcription factor IIIC contains a heterodimer resembling TFIIF Rap30/Rap74
Source: Nucleic Acids Res. 2013 Aug 5;41(19):9183–96. doi: 10.1093/nar/gkt664 (PMC3799434; doi:10.1093/nar/gkt664)
Supplement: Supplementary Data [file supp_gkt664_nar-01147-m-2013-File009.pdf]

SUPPLEMENTAL INFORMATION

RNA polymerase III-specific general transcription factor IIIC contains a heterodimer resembling TFIIF Rap30/Rap74

Nicholas M.I. Taylor[1], Florence Baudin[1,2], Gudrun von Scheven[1] and Christoph W. Müller[1,*]

[1]European Molecular Biology Laboratory, Meyerhofstrasse 1, 69117 Heidelberg, Germany
[2]UJF-EMBL-CNRS UMI 3265, Unit of Virus Host-Cell Interactions, 38042 Grenoble Cedex 9, France

Correspondence to CWM (cmueller@embl.de).

Keywords: RNA polymerase III, TFIIIC, TFIIF, transcription initiation, DNA recognition, winged-helix domain
Running title: Structure of the *S. pombe* TFIIIC Sfc1/Sfc7 heterodimer

SUPPLEMENTAL METHODS

Expression and purification of Sfc1 and Sfc7 constructs

S. pombe Sfc1 and Sfc7 were co-expressed overnight at 18 °C in BL21 (DE3) RIPL *E. coli* cells. Cells were harvested by centrifugation, resuspended in lysis buffer (50 mM Tris-HCl pH 7.4, 500 mM NaCl, 20 mM imidazole, 2 mM β-mercaptoethanol, 4 mM $MgCl_2$, 20% glycerol, DNase, lysozyme and protease inhibitor complex) and lysed by homogenization. The lysate was centrifuged and incubated with Ni-NTA resin, followed by batch purification. Beads were washed with washing buffer (50 mM Tris-HCl pH 7.4, 150 mM NaCl, 20 mM imidazole, 2 mM β-mercaptoethanol), high salt washing buffer (containing in addition 1 M NaCl) and again washing buffer. Subsequently, the protein complex was eluted using buffers identical to the washing buffer but containing increasing amounts of imidazole (up to 250 mM). Fractions containing the protein complex were eluted, incubated with TEV protease, and concurrently dialysed overnight in a buffer containing 20 mM Tris-HCl, 150 mM NaCl and 2 mM β-mercaptoethanol. The sample was then again incubated with Ni-NTA beads, followed by a second batch purification using buffers identical to the dialysis buffer but containing increasing amounts of imidazole. Fractions containing the complex without the tag were pooled and diluted two-fold with dilution buffer (20 mM Tris-HCl pH 7.4, 50 mM NaCl, 10 mM DTT). Then, the sample was applied on a Mono Q column (GE Healthcare) pre-equilibrated with buffer A (20 mM Tris-HCl, 100 mM NaCl and 10 mM DTT), and eluted with a linear gradient from 100 mM to 1 M NaCl. Fractions were pooled and concentrated before application on an S200 gel filtration column (GE Healthcare) pre-equilibrated with buffer containing 10 mM Tris-HCl pH 7.4, 100 mM NaCl and 10 mM DTT. The protein was then concentrated to 10 g/l. Limited proteolysis was performed using trypsin and chymotrypsin[51], and fragments were identified with MALDI peptide mass fingerprinting and electrospray ionization. Purification of Sfc1-NT/Sfc7 interaction module and $Sfc1_{186-456}$ constructs followed initially the same procedure as described for Sfc1/Sfc7. After the 2nd Ni-NTA chromatography step, fractions containing the complex were diluted sixfold with dilution buffer containing no NaCl (20 mM Tris-HCl pH 7.5, 10 mM DTT), followed by chromatography on a Q sepharose column (GE Healthcare) and elution with NaCl gradient (25 mM - 1 M NaCl). No gel filtration was performed. Wild type and mutant $Sfc1_{186-396}$ were purified similarly to the purification of the Sfc1/Sfc7 minimal interaction module constructs, except that the sample was only diluted three times after the second Ni-NTA chromatography step, and that a SP sepharose column (GE Healthcare) was employed. Protein was eluted using a linear

NaCl gradient (50 mM-1 M NaCl). Purification of $Sfc1_{111\text{-}396}$ was essentially the same. $Sfc1_{186\text{-}396}$ and $Sfc1_{111\text{-}456}$ were also purified using a two-step IMAC protocol, but a Q sepharose column was used instead of an SP sepharose column. $Sfc4_{129\text{-}537}$ was purified by glutathione affinity purification followed by S200 gel filtration. *S. pombe* τA (Sfc1/Sfc4/Sfc7) and τA lacking the C-terminal tail ($Sfc1_{1\text{-}396}$/Sfc4/Sfc7) was purified by glutathione affinity purification.

DNA binding oligonucleotides (A- and B-box underlined)

A-box (25 nt)	5'-CGAAG<u>TGGCCGAGTGGTCTA</u>TGGCG-3'
B-box (25 nt)	5'-CGACCCGG<u>GTTCGATTC</u>CCGGCCAA-3'
Non-specific (35 nt)	5'-CATGTATTGAACAGCGACTCGGGTTATGTGATGGA-3'
Glu1 tDNA (75 nt, 250 nt in total)	5'-TCCGATA<u>TAGTGTAACGGCTA</u>TCACATCACGCTTTCAC CGTGGAGACCGGG<u>GTTCGACTC</u>CCCGTATCGGAGCCA-3
